# *N*-(2-Oxo-2*H*-chromen-3-yl)cyclo­hexane­carboxamide

**DOI:** 10.1107/S1600536812047903

**Published:** 2012-11-24

**Authors:** Maria J. Matos, Lourdes Santana, Eugenio Uriarte

**Affiliations:** a Department of Organic Chemistry, University of Santiago de Compostela, Santiago de Compostela, Spain

## Abstract

In the title compound, C_16_H_17_NO_3_, the coumarin moiety is essentially planar [maximum deviation from the mean plane formed by the C and O atoms of the coumarin = 0.0183 (12) Å] and that the cyclo­hexane ring adopts the usual chair conformation. The dihedral angle between the mean plane of the coumarin residue and the plane of the amide residue (defined as the N, C and O atoms) is 18.9 (2)°. There are two intra­molecular hydrogen bonds involving the amide group. In one, the N atom acts as donor to the ketonic O atom and in the other, the amide O atom acts as acceptor of a C—H group of the coumarin. In the crystal, mol­ecules are linked into inversion dimers by pairs of N—H⋯O contacts and these dimers are linked into pairs by weak C—H⋯O hydrogen bonds. The combination of these inter­actions creates a chain of rings which runs parallel to [2-10]. C—H⋯π and π–π [centroid–centroid distance = 3.8654 (10) Å] inter­actions are also observed.

## Related literature

For the synthesis of the title compound, see: Viña, Matos, Ferino *et al.* (2012 ▶); Viña, Matos, Yáñez *et al.* (2012 ▶). For the biological activity of coumarin derivatives, see: Borges *et al.* (2009 ▶); Matos *et al.* (2009 ▶, 2010 ▶); Matos, Santana *et al.* (2011 ▶); Matos, Terán *et al.* (2011 ▶). For graph-set analysis of hydrogen bonds, see: Bernstein *et al.*, (1995 ▶)
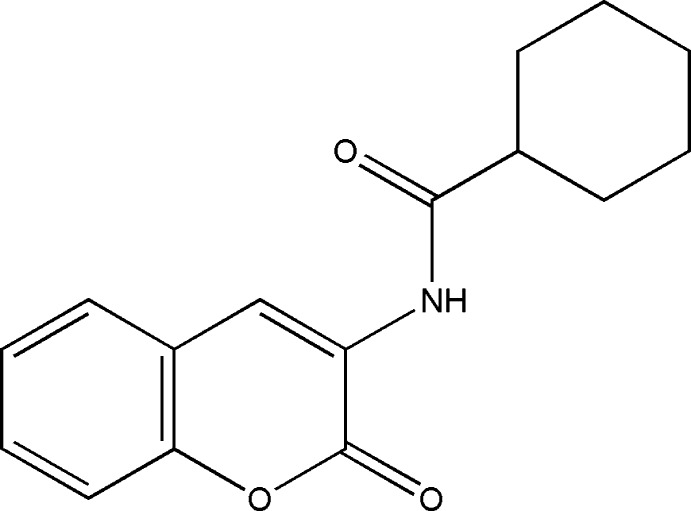


## Experimental

### 

#### Crystal data

C_16_H_17_NO_3_
*M* *_r_* = 271.31
Triclinic, 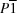

*a* = 6.4486 (6) Å
*b* = 9.6324 (11) Å
*c* = 11.0837 (11) Å
α = 83.061 (6)°
β = 89.134 (5)°
γ = 73.987 (5)°
*V* = 656.79 (12) Å^3^
*Z* = 2
Mo *K*α radiation
μ = 0.10 mm^−1^
*T* = 100 K
0.48 × 0.45 × 0.09 mm

#### Data collection

Bruker X8 APEXII KappaCCD diffractometer
Absorption correction: multi-scan (*SADABS*; Bruker, 2012 ▶) *T*_min_ = 0.910, *T*_max_ = 1.000
9698 measured reflections
2487 independent reflections
1834 reflections with *I* > 2σ(*I*)
*R*_int_ = 0.044

#### Refinement

*R*[*F* ^2^ > 2σ(*F* ^2^)] = 0.045
*wR*(*F* ^2^) = 0.113
*S* = 1.06
2487 reflections
185 parameters
H atoms treated by a mixture of independent and constrained refinement
Δρ_max_ = 0.20 e Å^−3^
Δρ_min_ = −0.25 e Å^−3^

### 

Data collection: *APEX2* (Bruker, 2012 ▶); cell refinement: *SAINT* (Bruker, 2012 ▶); data reduction: *SAINT*; program(s) used to solve structure: *SIR97* (Altomare *et al.*, 1999 ▶); program(s) used to refine structure: *SHELXL97* (Sheldrick, 2008 ▶); molecular graphics: *PLATON* (Spek, 2009 ▶); software used to prepare material for publication: *WinGX* (Farrugia, 2012 ▶).

## Supplementary Material

Crystal structure: contains datablock(s) global, I. DOI: 10.1107/S1600536812047903/go2076sup1.cif

Structure factors: contains datablock(s) I. DOI: 10.1107/S1600536812047903/go2076Isup2.hkl

Supplementary material file. DOI: 10.1107/S1600536812047903/go2076Isup3.cml

Additional supplementary materials:  crystallographic information; 3D view; checkCIF report

## Figures and Tables

**Table 1:** Hydrogen-bond geometry (Å, °) *Cg*1 and *Cg*2 are the centroids of the O1/C2–C5/C10 and C5–C10 rings, respectively.

*D*—H⋯*A*	*D*—H	H⋯*A*	*D*⋯*A*	*D*—H⋯*A*
N12—H12⋯O11	0.87 (2)	2.346 (19)	2.6990 (18)	104.7 (14)
N12—H12⋯O11^i^	0.87 (2)	2.098 (18)	2.9303 (16)	160.8 (17)
C4—H4⋯O14	0.95	2.37	2.9094 (19)	115
C7—H7⋯O14^ii^	0.95	2.57	3.473 (2)	158
C16—H16*B*⋯*Cg*1^iii^	0.99	2.81	3.5732 (17)	134
C17—H17*B*⋯*Cg*2^iii^	0.99	2.70	3.5876 (19)	149

Symmetry codes: (i) 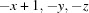; (ii) 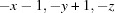; (iii) 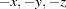.

**Fig. 1.**
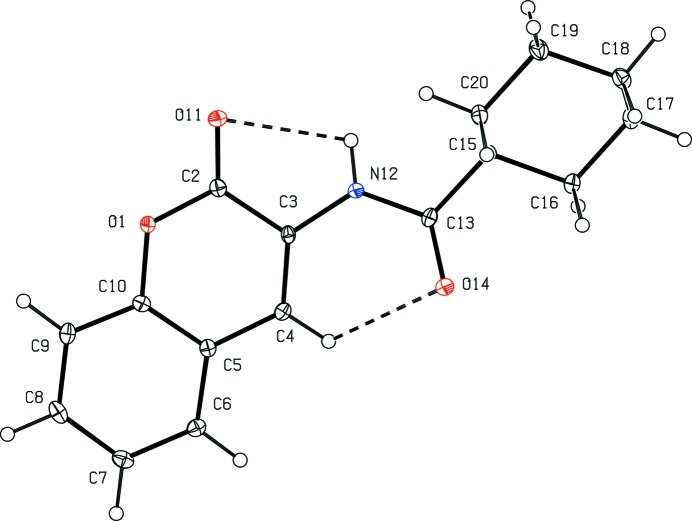
The molecular structure of the title compound with the atom-numbering scheme. Displacement ellipsoids are drawn at the 30%probability level. The dashed lines indicate intramolecular close contacts.

**Fig. 2.**
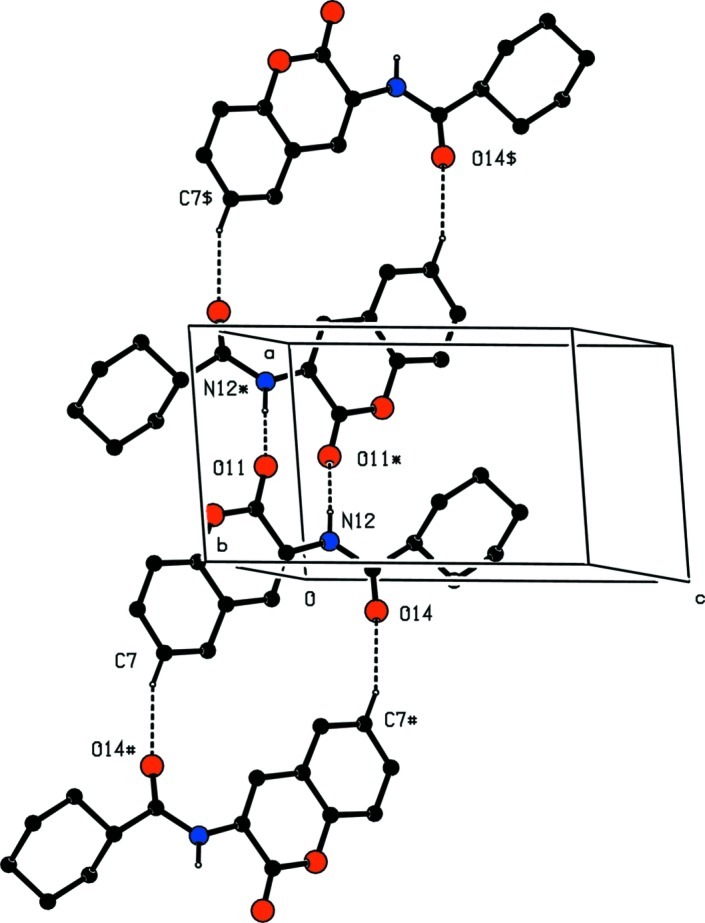
Part of the crystal structure of (2) showing the chain formed by molecules linked by R22(10) and R22(18) rings which runs parallel to the the {210]. Atoms labelled with an asterisk (*), hash (#) or dollar ($), are at(-x+1,-y,-z), (-x-1,-y+1,-z) and x+2,y-1,z respectively. Hydrogen atoms not involved in the hydrogen bonding have been omitted.

## supplementary crystallographic information

### Comment

Coumarin derivatives are very interesting molecules due to the biological properties that they may display (Borges *et al.* 2009; Matos *et al.* 2009, 2010; Matos, Santana *et al.*, 2011); Matos, Terán *et al.*, 2011). The title structure is a 3-substituted coumarin derivative that posses one cyclohexane ring linked by an amidic bridge at that position. Therefore, the X-ray analysis of this compound (figure 1) aims to contribute to the elucidation of structural requirements needed to understand the partial planarity of the compound (coumarin nucleus) and the torsion of the 3-substituent. Also, the X-ray analysis allows understanding the chair conformation of the cyclohexane. From the single-crystal diffraction measurements it can be concluded that the experimental bond lengths are within normal values with the average the molecule bond lengths. The planarity of the coumarin moiety is also evident by the torsion angles values between their carbons and oxygen atoms. The torsion angles C4—C3—N12—C13 (-21.3°), C3—N12—C13—C15 (-173.68°) and N12—C13—C15—C16 (-162.13°) are typical of the torsion permitted by the rotation of the amidic group at position 3. Also, the torsion angle C15—C16—C17—C18 (56.43°) is typical of a chair conformation of a cyclohexane ring.

There are intramolecular short contacts N12-H12···011 and C4–H4···O14.

The molecules are linked to form centrosymmetric R^2^_2_(10) dimers, (Bernstein *et al.*, 1995), by the N12-H12···O11(-x+1,-y,-z) hydrogen bond. The molecules are also link into R^2^_2_ pairs, (Bernstein *et al.*, 1995), by the weak C7···H7···O14 (-x-1,-y+1,-z) hydrogen bond.

Combination of these pair of interactions creates a chain of rings which runs parallel to [210]

There are C–H···π interactions between C16 and C17 and the centroids of the rings containing O11 and C9 respectively at (-x,-y,-z).

In addition there is π–π stacking between the rings containg O1 at (x,y,z) and (-x,-y+1,-z) in which the centroid to centroid distance is 3.8654 (10)Å, the perpendicular distance between the rings is 3.4428 (6)Å and the offset is 1.757Å.

### Experimental

*N*-(coumarin-3-yl)cyclohexanecarboxamide was prepared according to the protocol described by (Viña, Matos, Ferino *et al.* 2012; Viña, Matos, Yáñez *et al.* 2012). To a solution of 3-aminocoumarin (1 mmol) and pyridine (1.1 mmol) in dichlorometane (9 ml), the corresponding acid chloride (1.1 mmol) was added dropwise and the reaction was stirred, at room temperature, for 3 h. The solvent was evaporated under vacuum and the dry residue was purified by FC (hexane/ethyl acetate 9:1). A pale yellow solid was obtained in a yield of 72%. Suitable crystals for X-ray studies were grown from slow evaporation from acetone/ethanol: Mp 180–181 °C.

### Refinement

Refinement of *F*^2^ against ALL reflections. The weighted *R*-factor *wR* and goodness of fit *S* are based on *F*^2^, conventional *R*-factors *R* are based on *F*, with *F* set to zero for negative *F*2. The threshold expression of *F*^2^ > 2σ(*F*^2^) is used only for calculating *R*- factors(gt) *etc*. and is not relevant to the choice of reflections for refinement. *R*-factors based on *F*2 are statistically about twice as large as those based on *F*, and *R*- factors based on ALL data will be even larger.

### Crystal data

**Table:**

C_16_H_17_NO_3_	*F*(000) = 288
*M**_r_* = 271.31	F(000) = 288
Triclinic, *P*1	*D*_x_ = 1.372 Mg m^−^^3^
Hall symbol: -P 1	Melting point: 100 K
*a* = 6.4486 (6) Å	Mo *K*α radiation, λ = 0.71073 Å
*b* = 9.6324 (11) Å	Cell parameters from 1680 reflections
*c* = 11.0837 (11) Å	θ = 2.7–26.3°
α = 83.061 (6)°	µ = 0.10 mm^−^^1^
β = 89.134 (5)°	*T* = 100 K
γ = 73.987 (5)°	Plate, colourless
*V* = 656.79 (12) Å^3^	0.48 × 0.45 × 0.09 mm
*Z* = 2	

### Data collection

**Table:**

Bruker X8 APEXII KappaCCD diffractometer	2487 independent reflections
Radiation source: fine-focus sealed tube	1834 reflections with *I* > 2σ(*I*)
Graphite monochromator	*R*_int_ = 0.044
ω and phi scans	θ_max_ = 25.7°, θ_min_ = 1.9°
Absorption correction: multi-scan (*SADABS*; Bruker, 2012)	*h* = −7→7
*T*_min_ = 0.910, *T*_max_ = 1.000	*k* = −11→11
9698 measured reflections	*l* = 0→13

### Refinement

**Table:**

Refinement on *F*^2^	Primary atom site location: structure-invariant direct methods
Least-squares matrix: full	Secondary atom site location: difference Fourier map
*R*[*F*^2^ > 2σ(*F*^2^)] = 0.045	Hydrogen site location: inferred from neighbouring sites
*wR*(*F*^2^) = 0.113	H atoms treated by a mixture of independent and constrained refinement
*S* = 1.06	*w* = 1/[σ^2^(*F*_o_^2^) + (0.0577*P*)^2^] where *P* = (*F*_o_^2^ + 2*F*_c_^2^)/3
2487 reflections	(Δ/σ)_max_ < 0.001
185 parameters	Δρ_max_ = 0.20 e Å^−^^3^
0 restraints	Δρ_min_ = −0.25 e Å^−^^3^

### Special details

**Table:**

Experimental. ^1^H NMR (300 MHz, CDCl_3_): δ 1.27–1.95 (10H, m, 2H-2, 2H-3, 2H-4, 2H-5, 2H-6), 2.33–2.40 (1H, m H-1), 7.25–7.29 (2H, m, H-6, H-8), 7.33 (1H, dd, H-7, J=8.5, J=1.5), 7.46 (1H, dd, H-5, J=8.5, J=1.6), 8.12 (1H, s, H-4), 8.70 (1H, s, –NH); ^13^C NMR (75.47?MHz, CDCl3): δ 25.6, 25.7, 29.7, 43.0, 116.6, 120.2, 123.4, 124.3, 125.4, 128.0, 129.8, 150.1, 159.2, 175.9; DEPT: 25.6, 25.7, 29.7, 43.0, 116.6, 120.2, 124.3, 125.4, 128.0; MS m/z 272 ([M + 1]+, 16), 271 (M+, 100). Anal. Calcd for C16H17NO3: C, 70.83; H, 6.32. Found: C, 70.85; H, 6.35.
Geometry. All s.u.'s (except the s.u. in the dihedral angle between two l.s. planes) are estimated using the full covariance matrix. The cell s.u.'s are taken into account individually in the estimation of s.u.'s in distances, angles and torsion angles; correlations between s.u.'s in cell parameters are only used when they are defined by crystal symmetry. An approximate (isotropic) treatment of cell s.u.'s is used for estimating s.u.'s involving l.s. planes.
Refinement. Refinement of *F*^2^ against ALL reflections. The weighted *R*-factor *wR* and goodness of fit *S* are based on *F*^2^, conventional *R*-factors *R* are based on *F*, with *F* set to zero for negative *F*^2^. The threshold expression of *F*^2^ > 2σ(*F*^2^) is used only for calculating *R*-factors(gt) *etc*. and is not relevant to the choice of reflections for refinement. *R*-factors based on *F*^2^ are statistically about twice as large as those based on *F*, and *R*- factors based on ALL data will be even larger.

### Fractional atomic coordinates and isotropic or equivalent isotropic displacement parameters (Å^2^)

**Table:**

	*x*	*y*	*z*	*U*_iso_*/*U*_eq_	
O1	0.24398 (15)	0.35744 (12)	−0.13872 (9)	0.0151 (3)	
C2	0.2812 (2)	0.23542 (18)	−0.05704 (14)	0.0135 (4)	
C3	0.0969 (2)	0.20233 (18)	0.00628 (14)	0.0124 (4)	
C4	−0.1049 (2)	0.28990 (18)	−0.01851 (13)	0.0136 (4)	
H4	−0.2245	0.2668	0.0216	0.016*	
C5	−0.1385 (2)	0.41752 (18)	−0.10531 (13)	0.0128 (4)	
C6	−0.3423 (2)	0.51517 (19)	−0.13683 (14)	0.0157 (4)	
H6	−0.4679	0.4963	−0.1007	0.019*	
C7	−0.3625 (3)	0.63721 (19)	−0.21886 (14)	0.0177 (4)	
H7	−0.5009	0.7028	−0.2381	0.021*	
C8	−0.1796 (3)	0.66474 (19)	−0.27396 (14)	0.0185 (4)	
H8	−0.1938	0.7492	−0.3307	0.022*	
C9	0.0224 (3)	0.56983 (18)	−0.24641 (14)	0.0165 (4)	
H9	0.1473	0.5875	−0.2843	0.02*	
C10	0.0385 (2)	0.44942 (18)	−0.16300 (14)	0.0135 (4)	
O11	0.46651 (16)	0.16189 (12)	−0.04220 (10)	0.0183 (3)	
N12	0.1571 (2)	0.07624 (16)	0.08823 (12)	0.0147 (3)	
H12	0.285 (3)	0.019 (2)	0.0806 (15)	0.028 (5)*	
C13	0.0426 (2)	0.03698 (18)	0.18526 (14)	0.0139 (4)	
O14	−0.13975 (16)	0.10822 (13)	0.20623 (10)	0.0219 (3)	
C15	0.1649 (2)	−0.09965 (18)	0.26441 (14)	0.0131 (4)	
H15	0.24	−0.1722	0.2095	0.016*	
C16	0.0122 (2)	−0.16661 (18)	0.34364 (14)	0.0160 (4)	
H16A	−0.0669	−0.0959	0.3977	0.019*	
H16B	−0.0948	−0.1882	0.2911	0.019*	
C17	0.1367 (2)	−0.30629 (19)	0.42045 (15)	0.0188 (4)	
H17A	0.0353	−0.3448	0.4735	0.023*	
H17B	0.2042	−0.3802	0.3664	0.023*	
C18	0.3111 (3)	−0.27969 (19)	0.49876 (15)	0.0201 (4)	
H18A	0.2425	−0.2148	0.5595	0.024*	
H18B	0.3954	−0.3732	0.5431	0.024*	
C19	0.4615 (3)	−0.21078 (19)	0.42127 (15)	0.0212 (4)	
H19A	0.5427	−0.2807	0.3672	0.025*	
H19B	0.567	−0.1889	0.4748	0.025*	
C20	0.3377 (2)	−0.07084 (19)	0.34427 (14)	0.0174 (4)	
H20A	0.4394	−0.0317	0.292	0.021*	
H20B	0.2681	0.0028	0.3981	0.021*	

### Atomic displacement parameters (Å^2^)

**Table:**

	*U*^11^	*U*^22^	*U*^33^	*U*^12^	*U*^13^	*U*^23^
O1	0.0132 (6)	0.0145 (7)	0.0169 (6)	−0.0041 (5)	0.0012 (5)	0.0019 (5)
C2	0.0175 (8)	0.0115 (10)	0.0127 (9)	−0.0048 (7)	0.0005 (7)	−0.0039 (7)
C3	0.0168 (8)	0.0117 (10)	0.0096 (8)	−0.0053 (7)	0.0014 (6)	−0.0015 (7)
C4	0.0145 (8)	0.0160 (10)	0.0117 (9)	−0.0058 (7)	0.0013 (7)	−0.0037 (8)
C5	0.0170 (8)	0.0126 (10)	0.0098 (9)	−0.0048 (7)	0.0007 (7)	−0.0039 (7)
C6	0.0153 (8)	0.0196 (11)	0.0125 (9)	−0.0044 (7)	0.0004 (7)	−0.0043 (8)
C7	0.0199 (9)	0.0175 (10)	0.0133 (9)	0.0000 (7)	−0.0041 (7)	−0.0037 (8)
C8	0.0290 (9)	0.0128 (10)	0.0137 (9)	−0.0060 (8)	−0.0040 (7)	−0.0007 (8)
C9	0.0207 (9)	0.0178 (10)	0.0138 (9)	−0.0099 (7)	0.0015 (7)	−0.0022 (8)
C10	0.0150 (8)	0.0136 (10)	0.0117 (9)	−0.0029 (7)	−0.0016 (6)	−0.0031 (7)
O11	0.0129 (6)	0.0174 (7)	0.0229 (7)	−0.0024 (5)	0.0014 (5)	−0.0005 (5)
N12	0.0126 (7)	0.0145 (8)	0.0149 (8)	−0.0011 (6)	0.0024 (6)	0.0005 (6)
C13	0.0157 (8)	0.0161 (10)	0.0123 (9)	−0.0079 (7)	0.0021 (7)	−0.0036 (7)
O14	0.0162 (6)	0.0218 (8)	0.0230 (7)	−0.0005 (5)	0.0046 (5)	0.0035 (6)
C15	0.0161 (8)	0.0118 (10)	0.0112 (8)	−0.0036 (7)	0.0008 (6)	−0.0014 (7)
C16	0.0182 (8)	0.0169 (10)	0.0148 (9)	−0.0080 (7)	0.0019 (7)	−0.0019 (8)
C17	0.0241 (9)	0.0178 (11)	0.0156 (9)	−0.0083 (7)	0.0029 (7)	−0.0011 (8)
C18	0.0248 (9)	0.0174 (11)	0.0165 (9)	−0.0041 (8)	−0.0006 (7)	0.0003 (8)
C19	0.0213 (9)	0.0221 (11)	0.0194 (10)	−0.0067 (8)	−0.0049 (7)	0.0029 (8)
C20	0.0192 (8)	0.0172 (10)	0.0165 (9)	−0.0075 (7)	−0.0011 (7)	0.0013 (8)

### Geometric parameters (Å, º)

**Table:**

O1—C2	1.3610 (19)	C13—O14	1.2220 (17)
O1—C10	1.3852 (17)	C13—C15	1.514 (2)
C2—O11	1.2115 (17)	C15—C16	1.531 (2)
C2—C3	1.462 (2)	C15—C20	1.537 (2)
C3—C4	1.3523 (19)	C15—H15	1
C3—N12	1.391 (2)	C16—C17	1.526 (2)
C4—C5	1.434 (2)	C16—H16A	0.99
C4—H4	0.95	C16—H16B	0.99
C5—C10	1.389 (2)	C17—C18	1.525 (2)
C5—C6	1.410 (2)	C17—H17A	0.99
C6—C7	1.373 (2)	C17—H17B	0.99
C6—H6	0.95	C18—C19	1.521 (2)
C7—C8	1.395 (2)	C18—H18A	0.99
C7—H7	0.95	C18—H18B	0.99
C8—C9	1.383 (2)	C19—C20	1.527 (2)
C8—H8	0.95	C19—H19A	0.99
C9—C10	1.374 (2)	C19—H19B	0.99
C9—H9	0.95	C20—H20A	0.99
N12—C13	1.370 (2)	C20—H20B	0.99
N12—H12	0.867 (17)		
			
C2—O1—C10	121.98 (12)	C13—C15—C20	111.68 (14)
O11—C2—O1	117.05 (14)	C16—C15—C20	110.15 (13)
O11—C2—C3	124.73 (15)	C13—C15—H15	107.8
O1—C2—C3	118.23 (13)	C16—C15—H15	107.8
C4—C3—N12	127.29 (15)	C20—C15—H15	107.8
C4—C3—C2	120.24 (15)	C17—C16—C15	111.00 (12)
N12—C3—C2	112.46 (13)	C17—C16—H16A	109.4
C3—C4—C5	120.11 (15)	C15—C16—H16A	109.4
C3—C4—H4	119.9	C17—C16—H16B	109.4
C5—C4—H4	119.9	C15—C16—H16B	109.4
C10—C5—C6	116.77 (15)	H16A—C16—H16B	108
C10—C5—C4	119.08 (14)	C18—C17—C16	111.32 (14)
C6—C5—C4	124.15 (15)	C18—C17—H17A	109.4
C7—C6—C5	121.11 (15)	C16—C17—H17A	109.4
C7—C6—H6	119.4	C18—C17—H17B	109.4
C5—C6—H6	119.4	C16—C17—H17B	109.4
C6—C7—C8	119.91 (15)	H17A—C17—H17B	108
C6—C7—H7	120	C19—C18—C17	111.01 (14)
C8—C7—H7	120	C19—C18—H18A	109.4
C9—C8—C7	120.39 (16)	C17—C18—H18A	109.4
C9—C8—H8	119.8	C19—C18—H18B	109.4
C7—C8—H8	119.8	C17—C18—H18B	109.4
C10—C9—C8	118.56 (15)	H18A—C18—H18B	108
C10—C9—H9	120.7	C18—C19—C20	111.67 (14)
C8—C9—H9	120.7	C18—C19—H19A	109.3
C9—C10—O1	116.42 (14)	C20—C19—H19A	109.3
C9—C10—C5	123.25 (14)	C18—C19—H19B	109.3
O1—C10—C5	120.33 (15)	C20—C19—H19B	109.3
C13—N12—C3	127.25 (13)	H19A—C19—H19B	107.9
C13—N12—H12	115.9 (12)	C19—C20—C15	110.64 (14)
C3—N12—H12	116.6 (12)	C19—C20—H20A	109.5
O14—C13—N12	122.46 (15)	C15—C20—H20A	109.5
O14—C13—C15	123.73 (14)	C19—C20—H20B	109.5
N12—C13—C15	113.80 (13)	C15—C20—H20B	109.5
C13—C15—C16	111.49 (12)	H20A—C20—H20B	108.1
			
C10—O1—C2—O11	179.93 (13)	C4—C5—C10—C9	−179.32 (15)
C10—O1—C2—C3	−0.1 (2)	C6—C5—C10—O1	−178.81 (13)
O11—C2—C3—C4	−178.64 (15)	C4—C5—C10—O1	1.3 (2)
O1—C2—C3—C4	1.4 (2)	C4—C3—N12—C13	−21.3 (3)
O11—C2—C3—N12	0.8 (2)	C2—C3—N12—C13	159.30 (15)
O1—C2—C3—N12	−179.14 (13)	C3—N12—C13—O14	5.0 (3)
N12—C3—C4—C5	179.33 (14)	C3—N12—C13—C15	−173.68 (14)
C2—C3—C4—C5	−1.3 (2)	O14—C13—C15—C16	20.2 (2)
C3—C4—C5—C10	0.0 (2)	N12—C13—C15—C16	−161.13 (14)
C3—C4—C5—C6	−179.90 (14)	O14—C13—C15—C20	−103.50 (18)
C10—C5—C6—C7	−1.3 (2)	N12—C13—C15—C20	75.17 (17)
C4—C5—C6—C7	178.56 (15)	C13—C15—C16—C17	178.50 (13)
C5—C6—C7—C8	1.1 (2)	C20—C15—C16—C17	−56.93 (18)
C6—C7—C8—C9	0.0 (2)	C15—C16—C17—C18	56.43 (18)
C7—C8—C9—C10	−0.7 (2)	C16—C17—C18—C19	−55.19 (18)
C8—C9—C10—O1	179.81 (13)	C17—C18—C19—C20	55.31 (19)
C8—C9—C10—C5	0.4 (2)	C18—C19—C20—C15	−56.28 (18)
C2—O1—C10—C9	179.36 (13)	C13—C15—C20—C19	−178.89 (13)
C2—O1—C10—C5	−1.2 (2)	C16—C15—C20—C19	56.65 (17)
C6—C5—C10—C9	0.6 (2)		

### Hydrogen-bond geometry (Å, º)

Cg1 and Cg2 are the centroids of the O1/C2–C5/C10 and C5–C10 rings, respectively.

**Table:**

*D*—H···*A*	*D*—H	H···*A*	*D*···*A*	*D*—H···*A*
N12—H12···O11	0.87 (2)	2.346 (19)	2.6990 (18)	104.7 (14)
N12—H12···O11^i^	0.87 (2)	2.098 (18)	2.9303 (16)	160.8 (17)
C4—H4···O14	0.95	2.37	2.9094 (19)	115
C7—H7···O14^ii^	0.95	2.57	3.473 (2)	158
C16—H16*B*···*Cg*1^iii^	0.99	2.81	3.5732 (17)	134
C17—H17*B*···*Cg*2^iii^	0.99	2.70	3.5876 (19)	149

Symmetry codes: (i) −*x*+1, −*y*, −*z*; (ii) −*x*−1, −*y*+1, −*z*; (iii) −*x*, −*y*, −*z*.
